# Rhythmic alternate prioritization in auditory feature-based attention

**DOI:** 10.1038/s41598-026-50726-5

**Published:** 2026-05-04

**Authors:** Burcu Bayram, Sude Sarayköylü, Ulrich Ansorge, Ulrich Pomper

**Affiliations:** 1 Department of Cognition, Emotion, and Methods in Psychology, Faculty of Psychology, University of Vienna, Liebiggasse 5, Vienna, 1010 Austria; 2 Vienna Cognitive Science Research Hub, University of Vienna, Vienna, Austria

**Keywords:** Auditory, Rhythmic processing, Feature-based, Attention, Alpha oscillations, Neuroscience, Psychology, Psychology

## Abstract

Accumulating evidence suggests that attentional resources are distributed in a rhythmically alternating fashion between several simultaneously competing inputs. Most past research on this topic has investigated the visual modality, with some evidence of rhythmic sampling in auditory spatial attention. Here, we tested whether rhythmic sampling also extends to the auditory feature domain. Twenty-five human participants performed a challenging feature-based auditory attention task. On each trial, we presented an auditory cue (high or low tone) followed by a variable delay (250–1000 ms) and a target (high or low tone) at detection threshold, either congruent or incongruent with the cue (50% probability). By analyzing performance as a function of the cue-target interval, we arrived at three main results. First, sensitivity fluctuated significantly at a rate of 8 to 10 Hz for both congruent and incongruent targets, in line with cyclical waxing and waning attention towards the two features. Second, sensitivity in congruent and incongruent trials fluctuated out-of-phase with each other, suggesting an alternating prioritization of the two features. Third, across subjects, larger phase differences between conditions were associated with faster response times, indicating that alternate sampling facilitates task performance. In summary, our results demonstrate attentional sampling in yet another task domain and corroborate the idea of cyclic information processing as a key mechanism underlying information selection in the brain.

## Introduction

By now, sub-second fluctuations in human attention-related performance have been demonstrated in a number of sensory- and task domains^1–3^. In one of the earliest reports, Landau and Fries^4^provided evidence that visual spatial attention samples sensory input at around 4–10 Hz, using a modified spatial cueing paradigm. Behaviourally, this mechanism is typically studied by first resetting attention towards a certain location or feature via a (spatial or non-spatial) cue, and subsequently presenting a target at many different, densely sampled time-points. The resulting performance time-course commonly shows repeating fluctuations – that is, oscillatory patterns in the theta and alpha frequency bands (4–7 Hz and 8–14 Hz, resp^5^.. Electrophysiological studies have linked these fluctuations to oscillatory neural activity, reflecting a pattern of waxing and waning neural excitability^6–10^. Whether a stimulus is presented at a low or high excitability phase of the neural oscillation thus affects the respective behavioural performance. For instance, Busch et al.^7^ observed that visual stimulus detection accuracy depends on the pre-stimulus phase of ~ 8 Hz neural oscillations (but see^11^ for a recent, preregistered study that failed to replicate this effect). Similarly, Samaha and Postle^12^ found that the temporal resolution of visual perception correlates with the speed of occipital alpha-band oscillations, with a higher frequency associated with a finer temporal resolution.

Importantly, when two (or more) targets are monitored simultaneously, attention seems to prioritize them in an alternating manner, resulting in an out-of phase pattern of the performance fluctuations at each target. This mechanism has been demonstrated for visual, auditory and tactile spatial attention^4,13,14^, for visual object- and feature-based attention^5,15^, and even for visual working memory retention^16^.

While most of the evidence for attentional sampling comes from the visual domain, recent studies have observed similar effects in the auditory modality as well^14,17–22^. For instance, Ho and colleagues^14^ asked participants to detect faint target tones presented to either the left or right ear. At a variable interval preceding each target, a cue was presented at the same or the opposite ear. The authors found that detection sensitivity and bias fluctuated at 6 Hz and 8 Hz, respectively. Moreover, the respective time-courses for the two ears were out-of-phase, suggesting an alternation of spatial sampling. Plöchl et al.^22^ have subsequently replicated this effect while concurrently recording electroencephalography (EEG). In line with the hypothesized impact of oscillatory neural phase on local information processing capacity, they observed that lateralized occipital alpha-band power showed a theta-rhythmic modulation, which mirrored and predicted the concurrent behavioral oscillations. However, beyond this spatial attentional sampling mechanism, it is currently unknown whether auditory feature-based attention also fluctuates in the theta- to alpha frequency range, and whether two simultaneously monitored auditory features are likewise prioritized in an alternating manner. Data supporting such a mechanism within the auditory modality would be especially interesting given the role of low-frequency (2–14 Hz) neural oscillations and rhythmic entrainment in human speech processing^23–25^, selective auditory attention in multi-speaker environments^26,27^, and potential respective applications such as in smart hearing aids^28,29^.

In the present study, we aimed at answering these open questions using an auditory feature-based cuing experiment. On each trial, we presented participants with one of two uninformative cue-tones, followed by a variable delay and a target-tone close to detection threshold. Target pitch was either congruent or incongruent with the cue. The reasons we used uninformative cues was that past studies have repeatedly demonstrated the manipulation of auditory feature-based attention via uninformative cues^30–34^, as well as that we wanted to yield an equal number of congruent and incongruent trials. We hypothesized (1) that target detection sensitivity, indicative of feature-based attention, would fluctuate over time in the theta- to alpha range; and (2) that sensitivity time-courses for congruent and incongruent trials would fluctuate out-of-phase with each other, suggesting alternating prioritization of the two features. In line with our hypotheses and the idea of attentional sampling, we observed significant fluctuations in detection performance at 8–10 Hz for both congruent and incongruent cues. Importantly, performance fluctuations were out of phase between the two conditions, and the amount of phase separation additionally predicted the overall response time.

## Materials and methods

### Participants

Twenty-six participants took part in the study in exchange for monetary compensation (10 €/h). One participant did not return for the second data collection session and was therefore excluded from further analyses. The sample size was based on previous attentional-sampling studies, many of which have included between 20 and 30 participants^15,16,35^.

The remaining 25 participants (nine males, *M*_*age*_ = 23.4, *SD*_*age*_ = 3.4) were naïve to the purpose of the experiment. All gave written informed consent, and the study was conducted in accordance with the standards of the Declaration of Helsinki. We further followed the Austrian Universities Act of 2002, which states that only medical universities or studies conducting applied medical research are required to obtain additional approval by an ethics committee. Therefore, no additional ethical approval was required for our study.

### Experimental setup and task

We ran the experiment using OpenSesame (version 3.2.8^36^;) on a Windows 7 computer. Participants sat at a desk in a dimly lit room in front of a computer screen, with their head supported by a chin- and head rest. The auditory stimuli were delivered through over-ear headphones (Panasonic RP-HT 256), controlled via the computer’s sound card. We verified that the stimulus timing was accurate in the range of +/− 3 ms, via an oscilloscope connected to the computer’s sound output.

**Fig. 1:**
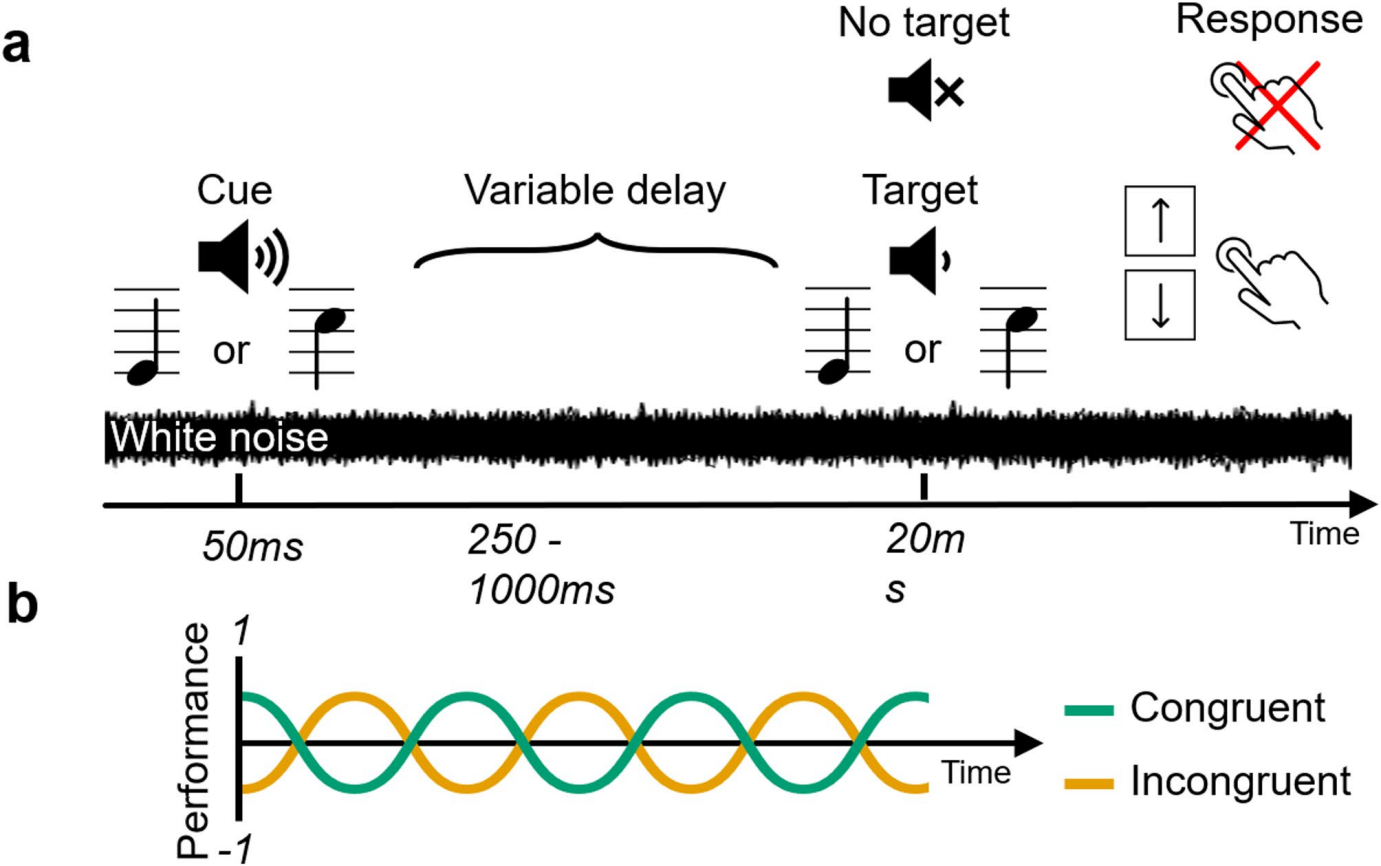
**(a)** Experimental design: At the beginning of each trial, participants received a noninformative auditory cue consisting of a high or low pitched pure tone. After a variable delay, an auditory target close to detection threshold was presented either at the cued or uncued pitch. Participants’ task was to indicate the target pitch (high or low) as fast and accurately as possible via a button press. To increase task difficulty, we presented white background noise throughout each trial (starting simultaneously with the cue). **(b)** Hypothesized results: We expected that target detection performance would cyclically fluctuate over time following the cue, and that performance for congruent and incongruent trials would be out-of-phase, corresponding to participants’ alternating monitoring of the two features (pitches).

Throughout the experiment, participants fixated a black dot at the center of a grey screen. At the start of each trial (see Fig. 1A), a clearly audible auditory cue (50 ms pure tone presented at 100% of the output volume in OpenSesame) of either a low or high pitch (440 or 800 Hz, respectively) was presented with equal probability (each 50%). We used noninformative cues, because rather than changing the overall focus of attention throughout a trial, the cue in our dense-sampling experiment was intended to reset feature-based attention to one pitch at a known time-point at the beginning of each trial, allowing to test for the presence of the hypothesized subsequent alternating cyclic shifts of attention between the two features. Past studies have effectively used noninformative cues^37^ to study the subsequent alternate sampling of two simultaneously monitored stimuli^4,13,22,38^. Starting from the cue-onset, we additionally presented white background noise throughout the trial until the response, to reduce the perceptual salience of the targets and thereby increase task difficulty. In other words, the noise acts as a simultaneous energetic masker, which raises the target discrimination thresholds by introducing broadband energy within the critical band around the tone frequency. The target sound levels were chosen such that the discrimination performance was difficult in the presence of the noise (on average 2.65% of the output volume in OpenSesame), but clearly above chance level (average *d*’ was 2.07 and 2.04 in the congruent and incongruent conditions, resp.). However, this was unlikely the case for the cue, which was presented at a clearly audible volume (100% of the output volume in OpenSesame) compared to the background noise (11% of the output volume in OpenSesame).

Next, we presented a variable delay of 250–1,000 ms varied in 10 ms steps, thus, resulting in 76 potential delay intervals. Crucially, testing detection performance at many different delay intervals following the noninformative cue allowed us to estimate the time-course of auditory attention. Finally, an auditory target (20 ms pure tone close to detection threshold) was presented either at the cued pitch (congruent trials) or at the uncued pitch (incongruent trials), each with 50% probability.

Participants reported a perceived target via a regular ‘QWERT’ keyboard, by pressing the ‘up’ arrow for high-pitched targets and the ‘down’ arrow for low pitched targets as accurately and fast as possible, using their right hand. We adaptively adjusted target stimulus intensity every six trials, separately for high- and low pitched targets, to yield a performance significantly above but close to 50% (within target-present trials), in order to keep the task demanding and maintain performance variability^5,35,39^. Specifically, target stimulus volume was automatically increased (decreased) by 0.5% of maximum sound volume in OpenSesame, if less (more) than four of the six previous target-present trials were correct. After each trial, participants received visual feedback on whether their response was correct or incorrect. At the end of each block, subjects received additional feedback regarding their mean hit-rate, false alarm rate, and response times (RTs).

Overall, we presented 10 trials per condition (congruent, incongruent) for each of the 76 variable delay intervals (i.e., a total of 1,520 trials). This number of trials has been shown to be sufficient for the detection of behavioral oscillations^16,35,39,40^. Additionally, we presented 300 trials containing no target to estimate the participants’ false-alarm rates. We explicitly informed participants about these trials and instructed them to only press a button if they were certain that a target had appeared. The order of trials was randomized across participants and self-timed breaks were administered every 56 trials. In total, the experiment comprised 1,820 trials, collected in two sessions on separate days.

Prior to the experiment, we assessed individual thresholds for the perception of the auditory target stimuli through an up-and-down staircase procedure of 64 trials and used the value of the final trial. The task design was identical to the main experiment. Stimulus intensity started out at a clearly perceivable level (5% of the output volume in OpenSesame and was reduced following each correct trial and increased following each incorrect trial (in steps of 0.5% of the maximal volume). As the task was identical to the main experiment, the staircase procedure also served as a practice block.

### Data analysis

We performed all data analyses using MATLAB (version 2018; The Mathworks, Natick, MA) and the CircStat toolbox (version 1.21^41^). First, we removed trials in which participants accidentally responded prior to the target (e.g., due to responding to the cue instead of the target; *M* = 5.2 trials; *SD* = 10.2). As a basic descriptive analysis, we computed mean sensitivity (*d‘*) and RTs (only for correct responses) separately for the congruent and incongruent conditions and compared performance between the conditions via paired-samples *t*-tests. Sensitivity (*d’*) was computed as the difference between the *z*-transformed hit-rate (i.e., correct responses to targets) minus the *z*-transformed false alarm rate (i.e., incorrect responses to no-target trials).

Next, to test our first hypothesis, we investigated the time course of sensitivity as a function of the cue-to-target delay interval, again separately for the congruent and incongruent conditions. To do so, we first sorted the trials for each participant according to their delay interval duration. Then, we used a moving-window averaging approach (step size of 10 ms) and computed the mean sensitivity within bins of five consecutive delay-period intervals (i.e., 50 ms in total per bin^16,22,39,42^, separately for the congruent and incongruent conditions. Subsequently, we detrended and normalized each single subject’s time course of sensitivity by subtracting the second order polynomial fit^5,14–16^ and performed fast Fourier transform (FFT) with a Hanning taper to estimate their spectral composition. For each subject, this yielded both power and phase values for 14 frequency bins from 1.3 Hz to 18.9 Hz.

To assess the statistical significance of spectral power in the performance time courses of sensitivity, we applied a non-parametric resampling procedure^4,5,16^. Specifically, we created 1,000 surrogate datasets under the null hypothesis that no periodic temporal pattern is present in the performance timeseries, by fitting each participant’s timeseries with an auto-regressive model with a single coefficient^43,44^:

$$\:{X}_{t}=\delta\:+{\varphi\:}_{1}{X}_{t-1}+{\epsilon\:}_{t}$$

Here, X_t_ denotes the value of the timeseries at time *t*, is a constant, _1_ is the autoregressive coefficient of the model that describes how the data at time *t −* 1 influence time *t*, and ε_t_ is a noise term. We then used this model to generate 1,000 surrogate time-courses for each condition and participant, with similar aperiodic properties to the original data, which we likewise transformed to the spectral domain via FFT^43^. Finally, for each condition and frequency band, we compared the empirical spectral power with the mean of the surrogate spectrum via *t*-tests (*p* =.05), and applied false-discovery rate (FDR) correction to compensate for multiple comparisons across frequency bands^45^.

To test our second hypothesis, we investigated potential phase differences between performance fluctuations for the congruent versus incongruent condition, as an indication of an alternating distribution of attention (i.e., alternating prioritized monitoring of the two pitches). To this end, for each significant frequency in the power-spectrum, we computed a common-median test (i.e., a non-parametric multi-sample test for equal medians, similar to a Kruskal-Wallis test for linear data^41^ between the congruent and incongruent condition’s phase angle. Similar to the spectral power analyses, these phase tests were conducted both on the empirical and the surrogate datasets, and statistical significance was assumed if the *p*-value from the empirical data exceeded 95% of *p*-values in the surrogate data (incl. FDR correction).

Finally, as an explorative validation of the functionality of the oscillatory fluctuations, we were interested in whether a larger phase difference between congruent and incongruent sensitivity timeseries is indicative of a better overall performance. To this end, for any potentially observed significant phase difference, we computed a correlation between each participant’s phase difference between conditions and their mean RT.

## Results

In the congruent condition, participants achieved an average sensitivity of 2.07 (*SD* = 0.48) and an RT of 601.0 ms (*SD* = 59.9). In the incongruent condition, average sensitivity was 2.04 (*SD* = 0.45) and RT was 599.5 ms (*SD* = 56.7). Neither sensitivity nor RTs differed between conditions, as revealed by *t*-tests (both *p*s > 0.51). The overall high sensitivity indicates that our participants were not guessing but adhered to the instructions of responding only when they were certain that a target had appeared. To rule out potential differences in stimulus intensity between congruent and incongruent trials due to our adaptive procedure, we additionally calculated pairwise *t*-tests between the respective target volume intensities. These revealed no indication for or against significant differences (low pitched sound: *t* = 1.82, *p* =.085, BF_10_ = 0.92; high pitched sound: *t* = 1.75, *p* =.095, BF_10_ = 0.85).

**Fig. 2:**
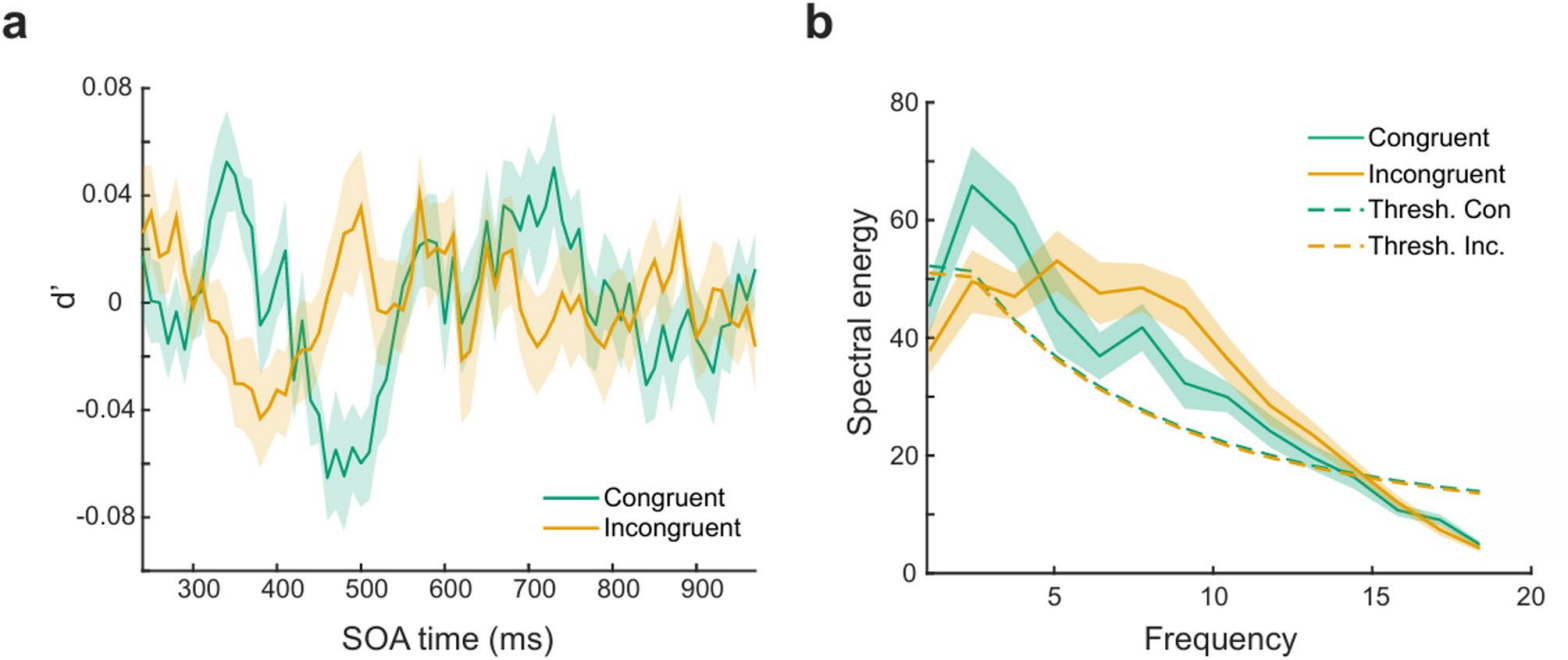
**a)** Grand average sensitivity (*d’*) for the congruent (green) and incongruent (yellow) condition as a function of the variable delay interval (here, stimulus onset asynchrony, SOA). Error ribbons show the *SEM*. **b)** Grand-average spectral energy of sensitivity fluctuations for the congruent (green) and incongruent (yellow) condition as function of their frequency. Empirical data is presented in solid lines, the significance threshold (thresh.) determined from resampled data in dashed lines. Error ribbons show the *SEM*.

**Fig. 3:**
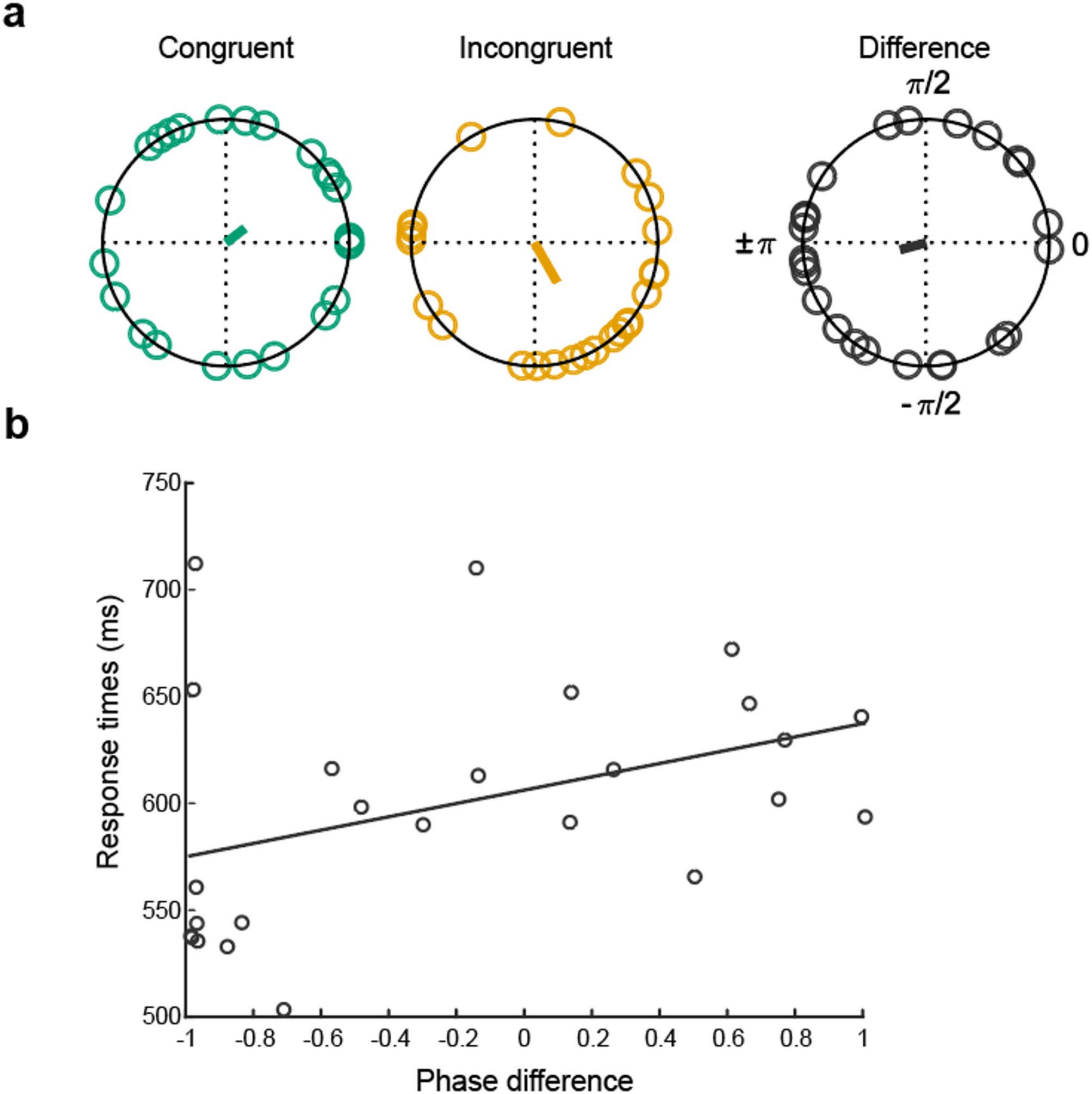
**(a)** Phase angles at 10.8 Hz for the congruent (left) and incongruent (middle) sensitivity time-series, as well as their differences (right). Small circles indicate individual participant’s angles or angle differences; lines represent mean angles or angle differences. **(b)** Correlation between individual sensitivity phase differences (between congruent incongruent conditions) and response times (across congruent and incongruent conditions).

In the participant’s average performance time courses (Fig. 2), we found that sensitivity fluctuates across time for both the congruent and incongruent conditions. Importantly, in line with our first hypothesis, these fluctuations exceeded chance level as shown via our spectral analysis (Fig. 3; Table 1). We observed significant spectral power between 2.7 Hz and 10.8 Hz for congruent trials and between 5.4 Hz and 13.5 Hz for incongruent trials.

Next, at 8.1 and 10.8 Hz, at which the spectral power was significant for both the congruent and incongruent condition, we tested whether the phase angles differed between conditions (Fig. 4a; Table 1). Confirming our second hypothesis, the common-median test indicated significant phase differences for sensitivity fluctuations at 10.8 Hz (*p* =.021), and this value further was significantly smaller than that of 95% of our 1,000 permutation datasets (*p* =.008). Notably, this phase effect is not confounded by power differences between the congruent and incongruent conditions at 10.8 Hz, *t*(30) = − 1.414, *p* =.170.

Finally, the correlation between the phase difference at 10.8 Hz and the overall RTs yielded a significant result (*r* =.402, *p* =.046), whose *p*-value was again smaller than in 95% of the surrogate data (*p* =.043), suggesting that a more pronounced counter-phase pattern of the sensitivity time-course between the congruent and incongruent conditions benefits behavioral performance (Fig. 3b).

**Table 1:**
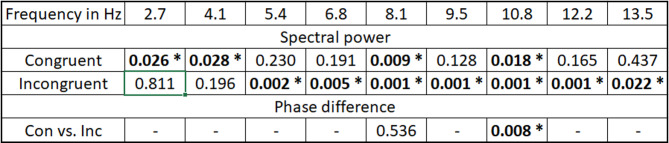
Statistical results (*p*-values) from the spectral-power (top) and phase-difference analysis (bottom) at each significant frequency (columns). Only frequencies containing at least one significant result are shown. Asterisks and bold font indicate significant datapoints (FDR corrected).

## Discussion

We investigated the presence of cyclic fluctuations in auditory feature-based attention using a behavioral dense sampling paradigm. In line with our hypotheses, we observed performance fluctuations at 8–10 Hz, indicative of a rhythmic sampling mechanism. Further, the phase difference in sensitivity time-courses between congruent and incongruent conditions suggests that the two task relevant features are prioritized in alternation. Finally, the extent of this phase separation correlates with overall RTs, corroborating its behavioral relevance.

### Feature-based auditory attention fluctuates at around 10 Hz

Basic auditory feature-based attention using noninformative cues is well documented in the literature^30–34^. For instance, over the course of four experiments, Green and McKeown (2001) observed increased detection performance for sounds whose pitch matched an uninformative previous cue compared to non-matching sounds. Additionally, the effect of the uninformative cue was only slightly below that of an informative one. Similarly, Alho et al. (2015) have observed BOLD activation in the same cortical areas following both endogenous- and exogenous attention, the latter elicited again via noninformative feature cues. Moreover, both Mondor et al. (1998) and Prime and Ward (2002) demonstrated that noninformative pitch cues not only lead to initially improved performance for targets with congruent pitches, but also to a subsequent inhibition of return with worse performance for congruent versus incongruent targets.

Here, we show that pitch-based performance is also subject to a much faster temporal fluctuation at around 8–10 Hz, mirroring sub-second performance fluctuations in visual, auditory and tactile spatial attention^4,13,14^ as well as visual object- and feature based attention^5,15^. This finding situates pitch-based auditory attention within the range of previously reported rhythmic sampling phenomena, while extending them to a feature-based domain in audition. Further, it corroborates the idea of cyclic information processing as a key mechanism in the brain, likely to rapidly and flexibly shift between several potential inputs of interest, as well as to deal with selection problems (e.g., keeping track of different features) or with limited resources^1,3^(for a recent account on the function and underlying neural mechanism of attentional sampling in the visual domain, see^46^.

Moreover, in addition to such spontaneous or ongoing performance fluctuations in the auditory domain, there is a large body of literature demonstrating the susceptibility of neural excitability and attention to external rhythmic entrainment, both to basic stimuli such as clicks or pure-tones^25,47,48^ and especially to speech^49,50^. For instance, Zoefel and VanRullen^50^ demonstrated that the detection of faint target tones depended on the phase of phonetic information in a concurrent speech stimulus, indicating that auditory attention can entrain to external rhythms defined not only by signal amplitude, but also by high-level attributes.

Interestingly, our observed sensitivity fluctuations are at a higher frequency than several of the previously reported examples. For instance, Ho and colleagues^14^tested auditory spatial attention and found oscillations in sensitivity at 6 Hz and in criterion at 8 Hz. Likewise, Plöchl et al^22^. observed 4–8 Hz performance oscillations in a similar auditory paradigm. Using a different task, Kayser^18^ had participants integrate auditory pitch changes over time and found rhythmic patterns in information integration at a frequency as low as 1–2 Hz (see also^20^. At the same time, studies have reported performance fluctuations in the higher alpha- and beta-band (10–20 Hz) frequencies as well, such as in motor-induced oscillations of response times^35^ or attentional sampling of tactile stimuli^13^. One reason for these differences in frequency could be the specific sensory modality and task, which, in our case (auditory and feature-based, respectively) differed from previous studies. In particular, the ~ 8–10 Hz fluctuation observed here may reflect task-specific demands associated with feature-based selection in audition. Further, a large body of research suggests separate processing streams for auditory spatial and feature information in dorsal and ventral cortical pathways, respectively^51–53^. Temporary representation of pitch information, thus, might recruit different functional oscillatory mechanisms than the auditory spatial information studied in attentional sampling experiments so far. Additionally, Chen et al^54^. have shown that task difficulty affects attentional sampling speed, with easier tasks resulting in higher sampling frequencies. Consequently, it is also possible that our present task was less challenging than many of the previously used ones, which is in line with our observed average sensitivity (*d’*) of 2.

Overall, these data indicate that ongoing rhythmic fluctuations in auditory performance can cover a wide array of frequencies, from the delta- up to the beta range (see also^3^, which can further entrain to external rhythms depending on the current task goals. In conjunction with our present data, this supports the notion that rhythmic sampling constitutes a general mechanism of attentional selection that extends to feature-based processing in audition, while being flexibly modulated by task demands and stimulus characteristics.

### Two simultaneously monitored features are prioritized in alternation

Going further, our data not only provide evidence for rhythmic sampling in general but show an out-of-phase pattern between the congruent and incongruent sensitivity time-course. As our participants were aware that the cues were noninformative and both targets were equally likely to appear, this suggests a task-specific alternating prioritization of two simultaneously attended features.

In a previous study in the visual domain, we likewise showed that when two templates are stored in visual working memory (WM), the ability to use these templates in a subsequent discrimination task fluctuates in antiphase between the templates^16^. Similar results of phase differences between the performance time-courses of concurrently attended-to or stored entities have previously also been reported for visual, auditory and tactile spatial attention^4,13,14^, as well as for visual object-based attention^5^(but see^15^ for no indication of phase difference in the case of visual feature based-attention).

Of particular relevance for the present study, Lui et al^21^. recently reported already tentative evidence for alternating sampling between two sounds of different pitches. Specifically, using a design related to our study, they observed that during a dual sound detection task, EEG activity evoked by both a low and a high frequency pitch depended on the pre-stimulus EEG phase in the range of 3–8 Hz. However, contrary to our findings, the respective phase angles for the low- and high-pitched sound did not differ from each other. Their interpretation is, thus, different from our present one, in that they assume two parallel processes oscillating in phase with each other, that simultaneously process auditory input at similar phases of the cycle, whereas we suggest a single oscillatory fluctuation that processes the two features at different phases of each cycle. Notably, while the study by Lui et al^21^. has the advantage of concurrent EEG recordings, the effect discussed above was only present in evoked responses and not in accuracy results, leaving the exact neural mechanism underlying the previous and current behavioral reports of alternate sampling still to be determined.

More generally, these findings tap into the ongoing debate on how many attentional templates can guide human behaviour at each point in time^55^.

While there is wide agreement that we can simultaneously hold multiple items in working memory (WM)^56^, on the one hand, several studies indicate that one of these items is in a prioritized state leading to increased behavioural relevance^57–60^. For example, participants in the study by Houtkamp and Roelfsema^58^ searched for either a single or two targets in a stream of visual objects. Performance was worse in the two-target compared to the single target condition, and worse than predicted by a model of two parallel template matching processes. On the other hand, research supporting the latter view promotes attentional guidance by several templates simultaneously^61–64^. Consistent with this interpretation, Kerzel and Witzel reported comparable attentional capture by distractors similar to the target, regardless of whether participants were instructed to search for one or two colors. These results imply that attention can be biased by more than one active feature template at a time.

To reconcile these two positions, we want to stress that we do not suggest a strict, all or nothing alternate sampling mechanism. While we speak of ‘attentional sampling’ in line with well-known previous literature, we assume cyclic changes in prioritization, such that the sensitivity towards a certain tone in- or decreases over time. In other words, we do not suggest that only one feature template can be active at a time, but that two templates may be concurrently activated in WM, with their respective gains alternating in a push–pull manner. In addition, whether these alternating gains represent a structural capacity limit or a way to strategically delineate efficient monitoring (e.g., to facilitate keeping track that each feature is monitored) remains to be determined^65^.

As we presented white masking noise throughout each trial, it is possible that the noise onset caused an additional attentional reset, on top of the reset via the cue. However, since its onset was simultaneously with the cue, it is not possible to isolate its potential reset effect on our presently analysed feature-based attentional fluctuations. Further, as our focus is on the effects of the pitch cues, which yielded differential results depending on whether the target pitch was congruent or incongruent, any additional effect of the noise is not central to our present research question.

### Feature phase-separation predicts overall RTs

As a final novel result, we observed a significant correlation between the sensitivity time-course phase difference of congruent versus incongruent conditions and the mean RTs. In other words, participants who showed a clearer, more pronounced pattern of alternate attentional switching between the two pitches also responded faster overall. Notably, this is a non-trivial finding: A mere correlation between sensitivity and RTs would have pointed toward an effect of vigilance or general attention, with participants with higher sensitivity also showing faster responses. Our finding, however, shows the more complex measure of phase difference between the two conditions being associated with facilitated task responses. A similar result has recently been observed in the visual domain^66^.

In detail, Xiong et al. reported rhythmicity at around 1 Hz for both target- and distractor processing during a visual motion detection task. Notably, a larger phase difference between the two rhythmic time courses was associated with higher target detection accuracy. Together, these data corroborate that when limited attentional resources need to be distributed between two potentially relevant inputs, performance is facilitated when those inputs are more separated in time within a sampling cycle.

## Conclusions

Rhythmic fluctuations in sensory information processing have now been established across several human sensory and task domains. In the present study, we demonstrated a similar mechanism underlying the processing of auditory feature information. Specifically, target detection sensitivity fluctuated rhythmically at 8–10 Hz, aligning well with neural alpha-band oscillations prominently associated with attentional gating. Further, detection performance for two simultaneously represented pitches fluctuated at different phase angles, suggesting that auditory attention cyclically alternates between features. Finally, the degree of this phase separation correlates with response speed across participants, further underscoring the behavioral relevance of this mechanism.

Together, our findings provide novel insight into the mechanisms of attentional sampling and auditory feature processing and corroborate the importance of cyclical information processing in the brain.

## Data Availability

The data that support the findings of this study are available under [https://osf.io/9dgva/files](https:/osf.io/9dgva/files).
